# Automatic Roadside Camera Calibration with Transformers

**DOI:** 10.3390/s23239527

**Published:** 2023-11-30

**Authors:** Yong Li, Zhiguo Zhao, Yunli Chen, Xiaoting Zhang, Rui Tian

**Affiliations:** 1 The Faculty of Information Technology, Beijing University of Technology, Beijing 100124, China; li.yong@bjut.edu.cn (Y.L.); zhaozhiguo@emails.bjut.edu.cn (Z.Z.); yunlichen@bjut.edu.cn (Y.C.); 2 The Faculty of Science Technology, Beijing University of Technology, Beijing 100124, China; zhangxiaoting@emails.bjut.edu.cn

**Keywords:** camera calibration, vanishing point detection, transformer

## Abstract

Previous camera self-calibration methods have exhibited certain notable shortcomings. On the one hand, they either exclusively emphasized scene cues or solely focused on vehicle-related cues, resulting in a lack of adaptability to diverse scenarios and a limited number of effective features. Furthermore, these methods either solely utilized geometric features within traffic scenes or exclusively extracted semantic information, failing to comprehensively consider both aspects. This limited the comprehensive feature extraction from scenes, ultimately leading to a decrease in calibration accuracy. Additionally, conventional vanishing point-based self-calibration methods often required the design of additional edge-background models and manual parameter tuning, thereby increasing operational complexity and the potential for errors. Given these observed limitations, and in order to address these challenges, we propose an innovative roadside camera self-calibration model based on the Transformer architecture. This model possesses a unique capability to simultaneously learn scene features and vehicle features within traffic scenarios while considering both geometric and semantic information. Through this approach, our model can overcome the constraints of prior methods, enhancing calibration accuracy and robustness while reducing operational complexity and the potential for errors. Our method outperforms existing approaches on both real-world dataset scenarios and publicly available datasets, demonstrating the effectiveness of our approach.

## 1. Introduction

In the domain of intelligent transportation systems, camera calibration [1,2,3] has been a perennial focal point of research due to its paramount importance in applying sensor data to assess system and device status, as well as perceiving the surrounding environment. This enduring focus is attributed to the paramount importance of precise camera parameter estimation, particularly in roadside scenarios where these parameters play a pivotal role in facilitating the transformation of 2D projections into essential 3D processes across various applications. This advancement in calibration is leading toward applications such as vehicle classification, counting, speed measurement, congestion detection, and more [2,3,4,5], where precise camera calibration is instrumental in enabling these functionalities and facilitates local projection compensation.

Calibration of roadside cameras is moving towards achieving the highest level of automation and minimizing manual interventions while enhancing calibration precision. With the development of intelligent transportation systems, the number of cameras has grown exponentially. Traffic scenes have distinct differences from other scenarios. In traffic scenes, traditional calibration boards [6] are no longer suitable because roadside cameras are often mounted at high angles, requiring large calibration patterns to meet calibration requirements. Additionally, it is not feasible to have all vehicles come to a stop for researchers to perform calibration. More importantly, in scenarios where PTZ cameras are installed or situations where camera parameters change due to external environmental factors affecting extrinsics or internal component variations affecting intrinsics, manual methods [7] are also unable to perform automatic parameter adjustments.

Currently, auto-calibration for roadside cameras mainly involves two methods: the vanishing point-based approach and the deep learning-based approach. In roadside scenes, there are primarily two categories of methods for vanishing point detection: the road-based vanishing point detection methods and the vehicle-based vanishing point detection methods The road-based vanishing point detection methods [8,9,10] involve detecting the first vanishing point location by detecting lane lines. Then, they combine the positions of other vanishing points, typically associated with objects like streetlights or cylindrical objects. Finally, the third vanishing point position is calculated using vector cross products.

These methods rely on clear and visible road markings for calibration, making them less suitable for scenarios where such markings are absent. The vehicle-based vanishing point detection methods [11,12] calculate the position of the first vanishing point based on the direction of the traffic flow. They then combine this with the calculation of the second vanishing point position using the edges of vehicles. Finally, relying on the Manhattan world assumption common in traffic scenes, the third vanishing point is calculated using cross products. These methods require precise detection of the second vanishing point and heavily rely on accurate estimation of edge directions. This process involves estimating edge directions by analyzing the shape of image gradient magnitudes (edge points) and applying threshold-based edge filtering. While these methods can achieve high accuracy, they are complex and leave room for improvement in terms of robustness. Additionally, these methods are less applicable in scenarios with curved roads, as they assume straight roads or nearly straight vehicle trajectories.

Deep learning-based roadside camera calibration methods [13,14,15] are not restricted to road scenes and do not require any manual input or prior information, making them increasingly popular in traffic scenarios. These methods are not limited by scene conditions and can provide relatively stable results. However, these methods lack certain geometric features and primarily extract semantic information from the scene, resulting in a decreased level of accuracy in scenarios with domain deviations from the training dataset.

Whether using traditional vanishing point detection methods or deep learning-based approaches, they either focus solely on vehicle features or on the features of the traffic scene, lacking a comprehensive consideration of both. This limitation hinders the improvement of camera calibration accuracy in traffic scenarios.

To address above issues and elevate calibration accuracy while building upon the foundation of automatic roadside camera calibration, we propose a camera calibration method based on Transformer for roadside vanishing point detection called “TLCalib”. Specifically, we introduce a unified segmentation descriptor encompassing the entire traffic scene, amalgamating vehicle optical flow tracking, vehicle edge segments, and scene line segments. This concurrently extracts vehicle trajectory features and traffic scene characteristics, augmenting feature diversity and representation. We incorporate pixel positions and angles in the line segment descriptor to represent their geometric features. Simultaneously, we perform feature sampling around the line segments to capture contextual attributes, which, when combined with pixel features, create a comprehensive line segment feature representation. This empowers the model to extract both geometric and semantic features in traffic scenes, enhancing its generalization capabilities by increasing feature dimensions and network representational capacity. To infer vanishing points, we employ a Transformer-based feature fusion module, enabling the clustering of line segments into three groups based on the positions of three vanishing points. This approach streamlines vanishing point detection by eliminating the need for edge-background models and manual parameter design. Once the line segments are clustered into these three groups, singular value decomposition (SVD) is used to compute the positions of the three vanishing points.

The key contributions of this paper are:

1. We have introduced a camera self-calibration model for vanishing point detection in traffic scenes called “TLCalib”, which is, to our knowledge, the first Transformer-based approach for this purpose. This model combines road and vehicle features to infer vanishing points, incorporating a broader range of features for enhanced accuracy. Additionally, it integrates both semantic and geometric features to improve adaptability across diverse scenarios.
2. To simplify the detection process for the second vanishing point while improving its accuracy, we adopt an approach that bypasses edge detection. Instead, we directly employ Line Segment Detector (LSD) to detect vehicle line segments in each frame and accumulate them. This process eliminates the need for designing edge-background models and utilizes the Transformer to predict line segment classification, achieving comparable results. This operation not only reduces manual parameter settings but also operational complexity and the potential for errors.
3. Our method has been tested in both real-world and dataset scenarios. Experimental results indicate that our approach outperforms traditional methods and deep learning-based methods in both traffic scenes and our dataset, achieving promising results.

## 2. Related Work

### 2.1. Transformers in Vision

The Transformer [16], originally introduced for machine translation [16], leverages multi-head self-attention and stacked feed-forward layers to capture extensive word interactions in sentences. Its robust representation capabilities have led to widespread adoption in various computer vision tasks. Dosovitskiy et al. [17] extend the Transformer paradigm to computer vision with the Vision Transformer (ViT), treating image patches as visual words and directly applying self-attention for image recognition. Carion et al. [18] deploy the transformer architecture (DETR) for end-to-end object detection. Transformers are also used in image super-resolution [19], camera calibration [20], video inpainting [21], tracking [22,23], and hold the potential to be applied in more applications. In our method, we apply Transformer encoder architecture in line segment clustering and scoring for vanishing point detection.

### 2.2. Camera Calibration

In the field of robotics [24] and vehicle applications [25], camera calibration is a widely explored domain due to the utmost importance of obtaining dependable geometric information from images in these areas. Currently, for monocular camera calibration, there are primarily four main categories of methods: Direct Linear Transform (DLT)-based, Zhang’s method, vanishing point-based, and deep learning-based approaches.

#### 2.2.1. Direct Linear Transform (DLT)-Based

From a fundamental perspective, camera calibration can be simplified as the task of finding a 3 × 4 homography matrix that encapsulates both the camera’s intrinsic parameters and the rotation between the camera and the world coordinate system. DLT typically does not yield the camera’s intrinsic and extrinsic parameters directly; instead, it provides a linear projection matrix. However, in certain downstream tasks, having the 2D-to-3D transformation relationship is sufficient for practical purposes. DLT method, initially proposed by Abdel-Aziz and Karara [7], is generally considered the most fundamental approach to this task. The DLT requires coordinates of at least four known corresponding points in both the image and the 3D scene. Subsequently, a 3 × 4 matrix can be obtained through the least-squares method. In many real-world scenarios, such as large-scale deployments in roadside perception, this approach proves to be less practical. This is primarily because the process of establishing correspondences between 3D world coordinates and 2D camera coordinates can be both time-consuming and labor-intensive, often necessitating meticulously crafted calibration setups and susceptible to human errors.

#### 2.2.2. Zhang’s Method

Zhang’s calibration method stands as the prevailing camera calibration technique in current practice, enjoying widespread recognition and adoption among both academic researchers and industry experts due to its elegant simplicity. This method only requires the presentation of the planar pattern from various camera orientations using a calibration board to determine the camera parameters with very high precision. However, this method is often unsuitable for traffic scenarios for several reasons. In traffic scenarios, the field of view is larger, and camera installation angles are higher, requiring larger calibration boards that are impractical for such scenarios. Furthermore, there is an increasing number of traffic cameras, making it unrealistic to use a calibration board for each camera. Lastly, it is not feasible to recalibrate cameras every time their parameters change in a traffic environment.

#### 2.2.3. Vanishing Point-Based

In situations where calibration boards are not readily accessible, and scene coordinate acquisition proves challenging, camera calibration methods leveraging vanishing point detection, as first introduced by Caprile and Torre in 1990 [26], have emerged as a crucial alternative. If there are multiple parallel lines in the scene, they will converge at a point when projected onto the image plane through perspective transformation. This point is referred to as the vanishing point, which can exist within the image or even beyond the image’s boundaries. In the field of computer vision, vanishing point detection has always been a fundamental and challenging problem. Mainstream vanishing point detection methods often rely on lines in the scene, primarily due to the fact that, under the Manhattan world assumption, lines naturally preserve information about the camera’s rotation, typically dividing the process into several stages. Typically, they begin with line segment detection [27,28]. Subsequently, the image is often set aside, and parametric line segments are clustered using techniques such as Hough transform [29], RANSAC [30,31], J-Linkage [32], EM algorithm [33], or dual space [34]. Calibration of the camera’s intrinsic parameters and rotation matrix can then be achieved using the vanishing points obtained from the camera view [35]. In many artificial environments, this method is highly practical since parallel lines are often present. However, due to the absence of scale information in vanishing points, it is not possible to determine the camera’s translation vector solely through this method.

#### 2.2.4. Deep Learning Based

On the contrary, in addition to the aforementioned three methods, recent neural network approaches utilize convolutional networks to learn semantic cues from input images and directly predict camera parameters. Liao et al. [36] have authored a comprehensive review that introduces deep learning-based camera calibration methods. Learning-based methods offer fully automatic camera calibration solutions that are applicable to any camera mounting angle and position, without manual intervention or calibration targets, distinguishing them from traditional methods. The authors mainly categorize methods for joint intrinsic and extrinsic calibration into two major classes: those based on geometric representations and those based on composite parameters. Geometric representation-based methods primarily involve vanishing points and horizontal lines.

**Vanishing Points**: DeepVP [37] was the first learning-based vanishing point detection method used to detect vanishing points from a single image. It disrupts the traditional process by scoring candidate horizons containing vanishing points. Chang et al. [38] reconfigured this task as a classification problem employing Convolutional Neural Networks (CNNs). They utilized an output layer that encompassed 225 distinct potential vanishing point positions. In the NeurVPS [39] framework, to bolster the efficacy of vanishing point detection, this methodology introduces an approach that leverages the geometric attributes of vanishing points, which manifest as the intersections of parallel lines, a canonical conic space and a conic convolution operator were presented, allowing for their utilization as standard convolutions within this specialized space. This approach endows the learning model with the capability to locally derive global geometric information concerning vanishing points. On the other hand, to improve the accuracy of the line segment clustering process, Tong et al. [40] proposed enhancing line segment clustering accuracy by combining image context, using a Transformer-based line segment classifier to group line segments in the image, and then estimating the corresponding vanishing points. The experimental results showed that incorporating image context can improve the accuracy of line segment clustering.

**Horizontal Lines**: The horizontal line is a essential contextual attribute in various computer vision tasks, especially in image measurement, computational photography, and 3D scene understanding. Projecting lines at infinity onto a plane perpendicular to the local gravity vector can determine the location of the horizon line. Given the camera’s field of view, pitch angle, and roll angle, it is easy to locate the horizon line in the captured image space. DeepHorizon [41] introduced the first learning-based method to estimate the horizon line from images without explicit geometric constraints or other clues. To train the network, they created a new benchmark dataset called Horizon Lines in the Wild (HLW), containing real-world images with annotated horizon lines.

**Composite Parameters**: As implied by its name, certain learning methods aim to concurrently acquire knowledge of both the camera’s intrinsic and extrinsic parameters. Hold-Geoffroy et al. [42], by jointly estimating composite parameters and training on a large-scale panoramic dataset [43], significantly surpassed previous independent calibration tasks. Their research findings indicate that these learning-based methods tend to focus on the intersections between the sky and the ground in scenes lacking prominent horizontal line features, such as clear and lengthy line segments. In doing so, neural networks often overlook characteristics of objects like clouds and trees. This behavior aligns with the way humans learn, as horizontal lines are indeed more likely to be found at the boundary between the sky and the land. As a result, such methods may experience a decrease in accuracy in scenes lacking the above-mentioned distinct features. Regarding learned feature categories, Lee et al. [44] and CTRL-C [20] simultaneously consider both semantic features and geometric cues for camera calibration. They demonstrated how utilizing geometric features can assist networks in understanding the potential perspective structure of images. Deep learning-based methods rely on extensive standard datasets and also need to consider domain adaptation in real-world scenarios. Therefore, they may experience accuracy drops in real-world scenes that have significant deviations from the training dataset.

### 2.3. Traffic Camera Calibration

In general, two attributes are crucial for roadside camera self-calibration: the algorithm’s ability to work automatically in any scenario without manual input for each installed camera and the algorithm’s suitability for cameras installed at various positions and angles relative to the road surface. Sochor et al. [5] have authored a comprehensive review introducing various traffic camera calibration methods. Kanhere and Birchfield [1] have also published a review on this topic, with a particular focus on commonly used vanishing point-based methods. These reviews provide valuable insights into the state of the art in traffic camera calibration techniques. Vanishing point-based methods have received the most in-depth research in traffic camera calibration. The common characteristic among nearly all these methods is the detection of vanishing points (VPs) that correspond to the direction of travel. In traffic scenes, methods for obtaining this VP primarily fall into two categories: road-based vanishing point detection and vehicle-based vanishing point detection.

#### 2.3.1. Road-Based Vanishing Point Detection

Common road scenes, such as road lines [9,45,46] lanes [46,47], and markings, often require a substantial number of traffic lanes with consistent and clearly visible lane markings for calibration. Pioneers in this field, such as Bas and Crisman [8], introduced a method that relies on manually marking two points on the road edges, along with known camera height and tilt angles. Calibration is performed by finding the vanishing point on the road’s edge points, assuming the road is straight. Zheng and Peng [9] explored the accuracy of similar manual methods for camera calibration. They also used two vanishing points, with the first one obtained from the ground on the lane markings and the second one from objects like lampposts in the vertical direction. Furthermore, they also explored solutions for scenarios where the camera’s principal point is not at the image center. This was achieved by incorporating the results of vanishing point estimation from multiple frames, enhancing the reliability of vanishing point detection, and improving the accuracy of camera calibration. Vanishing points were determined based on manual image annotations. The translation vector estimation was based on known lane and lane marking sizes and the known height of fences. A pioneering approach [10] was advanced to address the calibration challenges posed by roadside cameras. This approach harmoniously integrates top-down and bottom-up techniques to achieve self-calibration. In the bottom-up phase, the focus is on discerning road vanishing points, background lines, and vehicular travel zones. Meanwhile, the top-down process harnesses Markov Chain Monte Carlo (MCMC) for the sophisticated fitting and modeling of these ascertained features, encapsulating the intricacies of road geometry. These methods rely on manual or semi-manual processes and the availability of well-defined lane markings, making them suitable for certain road scenarios but limited in cases where road structures and markings are less clear or not present.

#### 2.3.2. Vehicle-Based Vanishing Point Detection

Due to the absence of stable and automatically detectable line markings in some scenarios, researchers have proposed several methods based on vehicle vanishing point detection. A prevalent assumption underlying this approach is the rectilinear alignment of vehicular traffic or the approximation of straight lanes. Subsequently, a detector is employed to identify vehicles [48], followed by the extraction of edge features through an edge detection mechanism [49]. Schoepflin and Dailey [50] pioneered an automated calibration technique. Their approach involved the utilization of a background model for initial lane identification, employing vehicle activity maps to establish lanes, and extracting a vanishing point from these lanes, operating under the assumption of lane linearity. Subsequently, they conducted statistical analysis utilizing the Hough transform on vehicle data to pinpoint a second vanishing point, this time oriented orthogonally. Leveraging a known lane width, they successfully achieved the calibration of all camera parameters. Dubska et al. [11] proposed a novel road-side camera self-calibration algorithm based on vehicle vanishing point detection. Building upon their previous work [51], this algorithm employed a cascaded Hough transform approach to detect the first vehicle vanishing point. Subsequently, an edge background model was developed to extract the edge features of moving vehicles, and the third vanishing point was calculated using a cross-product method. Sochor et al. [12] adopted Dubska’s vanishing point detection method but optimized the process of detecting the second vanishing point. They redesigned the edge background model and improved the scale inference process. These vehicle-based vanishing point detection methods leverage the motion and presence of vehicles as cues for estimating vanishing points. While they offer alternatives in scenarios lacking clear road markings, they may still require some level of user input or manual definition of lines. Furthermore, the accuracy of the final vanishing point is also to some extent dependent on the quality of the vehicle detection and tracking processes.

#### 2.3.3. Deep Learning-Based Camera Calibration in Traffic Scenes

Compared to the methods mentioned earlier, researchers are increasingly applying deep learning methods in traffic scenes. For instance, Bhardwaj et al. [13] proposed a relatively new automatic calibration method. They used deep learning to detect key points on vehicles, assuming relative sizes based on statistical data of the most common local car models. They assumed that the camera’s intrinsic parameters were known and assumed an appropriate rear-view perspective of the vehicles. The necessary vehicle key points included taillights and side mirrors. However, this method has limitations due to the need for prior knowledge of camera intrinsic information and reliance on the spatial structure of specific vehicle keypoints. Kocur et al. [14] proposed a Convolutional Neural Network (CNN) for vanishing point detection that outputs a heatmap, where the vanishing point’s position is represented using diamond space parametrization. This approach can detect vanishing points from the entire infinite projection space and, unlike previous methods, does not require the road scene to be straight or depend on specific vehicle types. However, it only identifies semantic features for the entire scene and does not utilize extensive geometric information, resulting in lower accuracy. Zhang et al. [15] introduced a deep calibration network that estimates vanishing points and camera extrinsic parameters from RGB images in an end-to-end manner. Then, they calculate the camera focal length using the vanishing point and camera rotation angle based on perspective projection geometry. Similar to Kocur’s [14] approach, this method directly learns vanishing point locations but lacks scene geometry information.

Summarizing the current state of roadside camera calibration methods, most of them either focus solely on learning simple scene features or vehicle-related features. Alternatively, they concentrate on either geometric features or semantic features.

Two main limitations give rise to challenges in further enhancing the accuracy of automatic calibration for traffic cameras. Methods in some scenarios emphasize vehicle features, while others focus on scene markings. This choice depends on the challenges posed by detecting traffic markings, which can be problematic or missing. Vehicle-related vanishing points offer an alternative in such cases. In contrast, methods solely detecting scene markings favor their stability in representing scene layout and contours. Moreover, they easily combine with existing features for scene scale inference. Recent research has shown that combining both geometric and semantic cues can significantly improve camera calibration accuracy. Geometric features provide interpretable constraints for vanishing points, enhancing their adaptability to domain shifts and improving the model’s generalization capabilities. Semantic features offer context associations for the scene, boosting the inference capacity of vanishing points. This empirical finding is equally applicable in traffic scenarios. Therefore, in this paper, we tackle the constraints of previous methods by utilizing a Transformer to jointly represent traffic scenes and vehicle characteristics, while also acquiring both geometric and semantic insights.

## 3. Auto Calibration Principle of Traffic Camera

**Figure 1:**
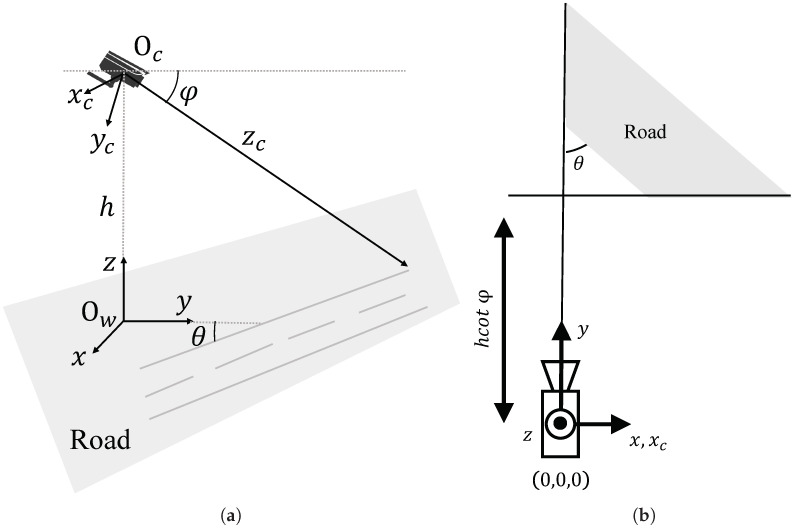
The camera space model: (**a**) diagram of the camera space model; (**b**) top view of the camera space.

Prior to elucidating the vanishing point estimation model employed in this article, we introduce contemporary camera calibration models [1]. Within this context, we streamline the camera as an optical model with its principal point situated at the image center. While this specification may not be a flawless fit in some scenarios, any potential discrepancies for subsequent tasks are not the central concern of this study. Additionally, the assumption of the imaging plane being orthogonal to the optical axis aligns with established conventions in previous traffic camera calibration models. Figure 1 illustrates the schematic diagram of the camera spatial model for road scenarios, with a right-handed coordinate system. For the sake of convenience in subsequent analysis, we set the camera’s focal length as *f*, the distance between the camera origin and the ground as *h*, and the camera’s pitch angle as φ. Since the roll angle of the camera in our scenario is typically very small, the errors in downstream tasks such as localization and speed estimation can be considered negligible. Consequently, these errors have minimal impact on the calibration results presented in this paper and are therefore not taken into account.

As shown in Figure 1a, this model defines the CCS $x_{c}y_{c}z_{c}$ and the WCS xyz. The CCS’s origin is located at the principal point on the image plane, with the $x_{c}$ and $y_{c}$ axes aligned with the image rows and columns, respectively, and the $z_{c}$ axis aligned with the camera’s optical axis. The WCS has its origin aligned with the projection of the CCS’s origin onto the ground plane, with the *x* axis parallel to $x_{c}$ and the *y* axis perpendicular to *x* and lying on the road plane. Considering the homogeneous representation of a point in 3D space as $x={\left[\begin{array}{c} x,y,z,1 \end{array}\right]}^{T}$, its transformation in the image coordinate system is $p={\left[\begin{array}{c} \alpha u,\alpha v,\alpha \end{array}\right]}^{T}$. Here, α≠0, and thus, the projection equation from the WCS x to the ICS p is given by:

(1) p=KRTx,

where K, R, and T, respectively, represent the intrinsic matrix, rotation matrix, and translation matrix. From the above analysis, it can be deduced that:

(2) $$K=\left[\begin{array}{ccc} f & 0 & 0\\ 0 & f & 0\\ 0 & 0 & 1 \end{array}\right],$$

(3) $$R=\left[\begin{array}{ccc} 1 & 0 & 0\\ 0 & -\sin\varphi & -\cos\varphi \\ 0 & \cos\varphi & -\sin\varphi \end{array}\right],$$

(4) $$T=\left[\begin{array}{cccc} 1 & 0 & 0 & 0\\ 0 & 1 & 0 & 0\\ 0 & 1 & 1 & -h \end{array}\right].$$

Substituting Equations (2)–(4) into Equation (1) yields the expanded projection model.

(5) $$\left[\begin{array}{c} \alpha u\\ \alpha v\\ \alpha \end{array}\right]=\left[\begin{array}{cccc} f & 0 & 0 & 0\\ 0 & -f\sin\varphi & -f\cos\varphi & fh\cos\varphi \\ 0 & \cos\varphi & -\sin\varphi & h\sin\varphi \end{array}\right]\left[\begin{array}{c} x\\ y\\ z\\ 1 \end{array}\right].$$

### Auto-Calibration Model

**Figure 2:**
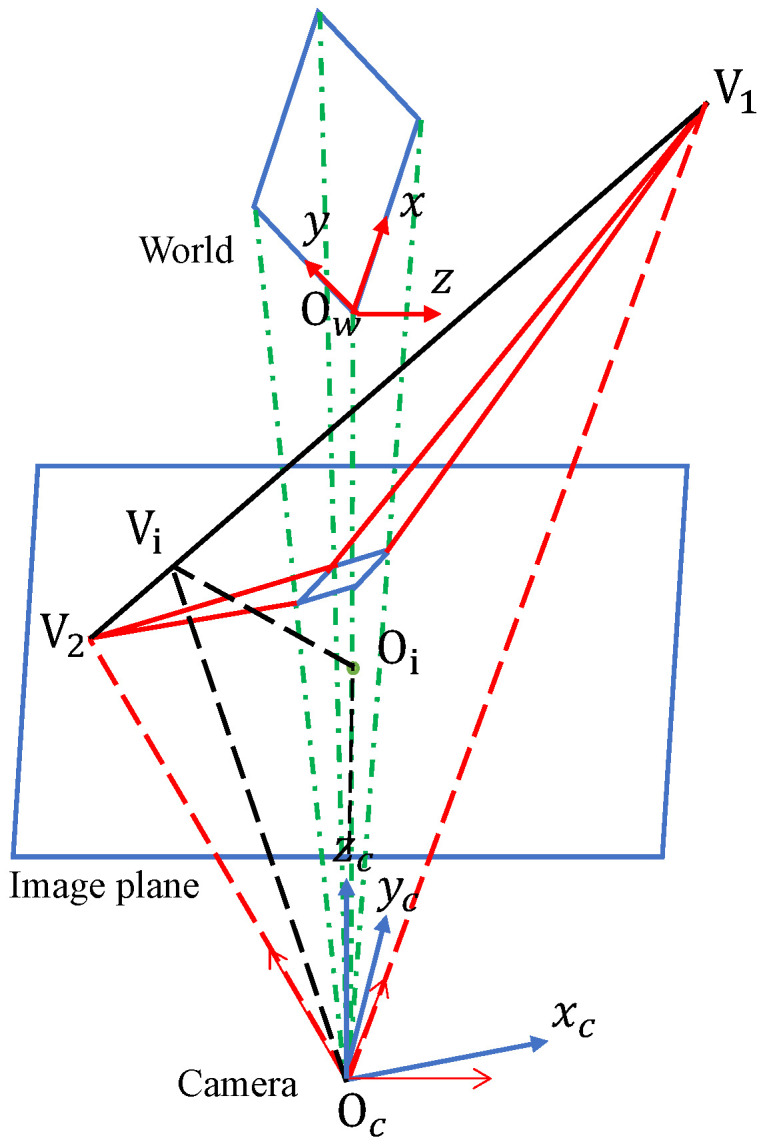
The schematic diagram of the vanishing point-based camera self-calibration model.

Expanding upon the previously posited assumptions, the camera’s self-calibration model, as illustrated in Figure 2, extends its utilization of vanishing points. In the context of conventional traffic road scenes conforming to the Manhattan world hypothesis, three prominent vanishing directions emerge: the lane direction, a direction perpendicular to the lane while parallel to the road surface, and a direction perpendicular to the road surface. It is well-established that possession of any two vanishing points facilitates the calculation of intrinsic and extrinsic camera parameters. The WCS, identical to Figure 1, has its origin at $O_{w}$, with mutually perpendicular *x*, *y*, and *z* axes. The CCS also matches Figure 1, with its origin at $O_{c}$, featuring mutually perpendicular $x_{c}$, $y_{c}$, and $z_{c}$ axes. The projection of $O_{c}$ on the image plane is denoted as $O_{i}$. $V_{1}$ and $V_{2}$ represent two vanishing points, with their coordinates on the ICS as $V_{1}=\left(u_{1},v_{1}\right)$, $V_{2}=\left(u_{2},v_{2}\right)$, and the projection of $O_{i}$ on the line $V_{1}V_{2}$ is denoted as $V_{i}$. The focal length *f* can be obtained by calculating the distance between $O_{c}$ and $O_{i}$ along the optical axis, i.e.,

(6) $$f=\left||O_{c}O_{i}|\right|=\sqrt{{\left||O_{c}V_{i}|\right|}^{2}-{\left||O_{i}V_{i}|\right|}^{2}},$$

where $O_{i}V_{i}$ represents the distance between the image center and the horizontal line, which is determined by the two vanishing points. Furthermore,

(7) $$\left||O_{c}O_{i}|\right|=\sqrt{\left||V_{1}V_{i}|\right|\cdot \left||V_{i}V_{2}|\right|}.$$

(8) $$\varphi =\tan^{-1}\left(\frac{-v_{1}}{f}\right).$$

The derivation of the camera’s intrinsic and extrinsic parameters is facilitated through the substitution of Equations (6)–(8) into Equation (5).

## 4. Vanishing Point Detection Network

### 4.1. Representation of the Vanishing Points

Traffic monitoring heavily relies on vehicle-related information. Compared to camera self-calibration methods solely relying on road markings or vehicle trajectories, combining both methods with line segment segmentation features can lead to more reliable vanishing point detection results, thereby further improving the camera calibration accuracy in traffic scenes. In this paper, we initially identify a variety of line segment features within the scene, encompassing lane markings, pedestrian pathways, streetlights, traffic signs, and more. These segments will be combined with vehicle trajectory features later and input together into the model for unified classification. For scene line segment detection, we can readily employ the LSD [28]. Despite being a traditional method, LSD exhibits a notably high level of detection accuracy and operates at a rapid pace, effectively meeting the demands of traffic scenarios.

**Figure 3:**
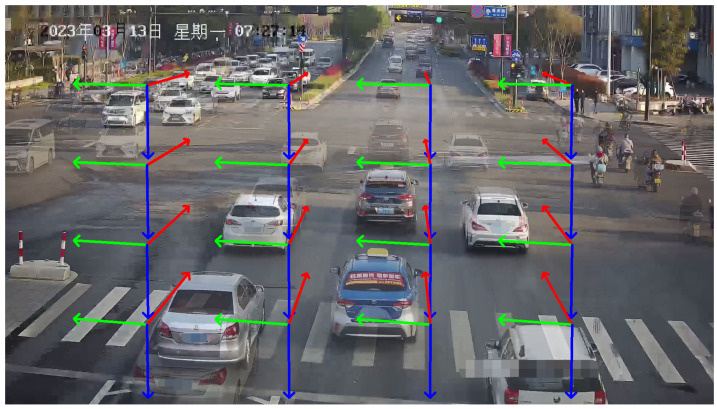
Illustration of three vanishing points in traffic scenarios. Three lines with red, green, and blue arrows illustrate the directions of the first, second, and third vanishing points.

Regarding vehicle trajectory features, they are typically divided into three orthogonal vanishing points based on the Manhattan assumption. Figure 3 illustrates the color-coded vanishing point and their corresponding directions. The first vanishing point, represented by the color red, corresponds to the direction of vehicle movement. The second vanishing point, indicated in green, is perpendicular to the direction of vehicle movement and lies parallel to the road surface. The third vanishing point, denoted by the color blue, is perpendicular to both the road surface and the direction of the first vanishing point.

To determine the location of the first vanishing point, we utilize vehicle trajectories constructed from optical flow tracking as indicators, like [11,12,52,53]. The steps of the method are as follows:

1. Firstly, we use an object detector to detect vehicles. Considering the inference speed for traffic scenes and real-time requirements for large-scale deployment, YOLO V7 [54] is employed, which offers high detection accuracy and FPS.
2. Feature points are extracted within the bounding boxes, and optical flow is employed to track the vehicles, obtaining a collection of trajectories, denoted as $T=\{T_{1},T_{2},\ldots ,T_{n}\}$, where $T_{i}=\left\{\left(u_{j},v_{j}\right),j=1,2,\ldots ,k\right\}$ represents the optical flow tracking results.
3. In real-world scenarios, roads have curved sections. To stably acquire the first vanishing point, it is necessary to eliminate trajectories in set *T* that do not satisfy linear conditions, i.e., for $\forall T_{i}$, $T_{i}=\left\{TP_{ik},k=1\ldots m\right\}$.
4. If some sets $TP_{ik}$ do not satisfy the linear conditions, they are excluded, resulting in $T_{i}^{*}$, and a new set of trajectories is obtained, denoted as $T^{*}=\left\{T_{1}^{*},T_{2}^{*},\ldots ,T_{n}^{*}\right\}$.
5. As shown in Figure 3: the direction indicated by the red lines corresponds to the first vanishing point.

Subsequently, these line segment trajectories $TP_{ik}$ are treated as part of the overall line segment detection process. Although deep learning-based sparse optical flow tracking algorithms offer better robustness to factors like illumination changes and noise, and higher accuracy, they require more computational resources and time during inference due to limitations in inference speed and image resolution. The inference accuracy of vanishing points may be significantly reduced when these methods are applied in practical scenes and combined with object detection and camera calibration models. So, similar to previous works [11,12,52,53], we use the Min-eigenvalue point detector [55] and the KLT tracker [56]. While acknowledging that it may be affected by certain lighting conditions, we address this aspect within the line segment classification model by cleverly scoring and clustering different lines to effectively mitigate the issue.

**Figure 4:**
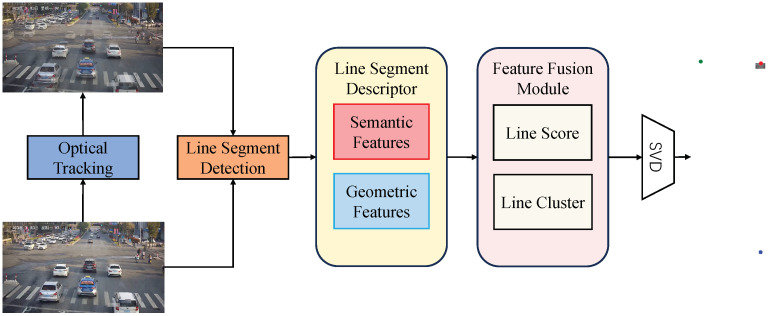
The overall architecture diagram of the vanishing point detection network in this paper.

After obtaining the reasoning features for the first vanishing point, we establish the features for the second vanishing point, denoted as V2. Unlike previous methods [11,12] that detect the second vanishing point using lines passing through vehicle edges, which often coincide with V2, this step heavily relies on the correct estimation of edge directions. It also requires analyzing the shape of image gradient magnitudes (edge points) to estimate edge directions from a larger neighborhood. Although these methods achieve higher accuracy, the process is intricate and leaves room for improvement in robustness. Therefore, In our approach, we directly use the LSD to detect vehicle line segments in each frame and then accumulate them, as shown in Figure 4. We avoid any image gradient calculations and edge direction estimation. Instead, we integrate this part together with the features of the first and third vanishing points into a unified model for end-to-end line classification and vanishing point estimation. On the one hand, our model can represent these segments as local image contexts containing their geometric features (i.e., direction and position). On the other hand, utilizing a feature fusion module based on the Transformer model, we systematically capture non-local correlations among all line segments, predict probabilities for each group, and estimate confidence scores for the vanishing points. Below, we will provide specific details of the model.

### 4.2. Line Segment Module

**Figure 5:**
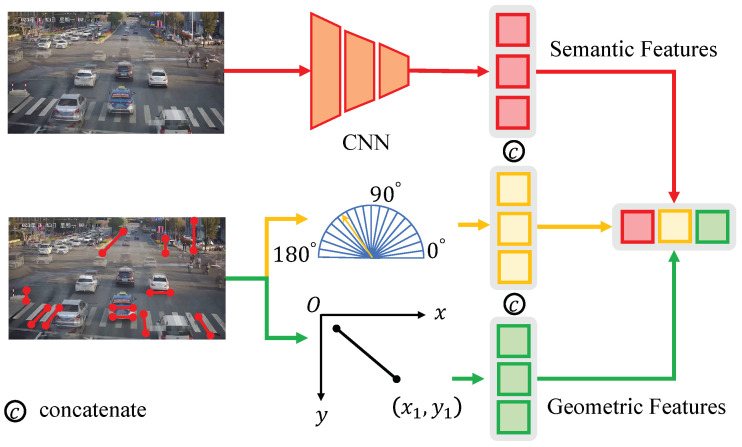
Line segment descriptor in vanishing point detection: the red lines in the bottom-left picture represent examples of line segment detection results.

As shown in Figure 5, A predefined 1D vector of a specific size is utilized to portray a line segment, where geometric characteristics and local image context are merged. Geometric features encompass the line segment’s position and orientation within the image. Position is determined through equidistant point sampling along the segment, while direction is represented using one-hot encoding within predefined intervals spanning from 0° to 180°.

In this module, we explore the utilization of semantic features in the vicinity of the line segment for description. Initially, feature maps are extracted from the image using a CNN model. Subsequently, for each line segment, we uniformly sample *N* points, producing a feature vector set of dimensions N×C. Given that the sampled points may not precisely match the feature map’s grid points, we employ bilinear interpolation to calculate the feature values for each sampled point based on the surrounding grid points. To attain a consistent 1D representation for each line segment, denoted as $f_{con}$, we conclude the process by conducting a weighted summation of the feature vectors, resulting in a size of 1×C.

The ultimate representation of a line segment, denoted as $f_{line}\in R^{1\times C}$, is constructed by concatenating the three aforementioned features, expressed as follows:

(9) $$f_{line}=\left[f_{con},f_{pos},f_{dir}\right].$$

In the specific implementation, ResNet18, as detailed in reference [57], functions as the semantic feature extraction module, subsequently being succeeded by a 1 × 1 convolutional layer that serves the purpose of channel dimension reduction to 32. Position representation utilizes 32 coordinates, derived from the uniform sampling of 16 points. Direction representation is achieved through a one-hot vector with a size of 36.

### 4.3. Feature Fusion Module

Predicting group probabilities and confidence scores is the main function of our feature fusion module, which takes as input the feature vectors of the line segments. This module comprises two distinct network branches, designated as $T_{cluster}$ and $T_{score}$.

Using feature vectors of *M* line segments, represented as $f_{lines}\in R^{M\times C}$, $T_{cluster}$ estimates the probability *p* that each line segment is associated with an unidentified yet desired vanishing point, described as follows:

(10) $$p=softmax(T_{cluster}\left(f_{lines}\right)).$$

$T_{score}$ is employed to forecast the confidence score *s* for each line segment during the estimation of the vanishing point, represented as:

(11) $$s=sigmoid(T_{score}\left(f_{lines}\right)).$$

A greater confidence score implies an augmented likelihood of the line segment intersecting with or being in close proximity to the vanishing point.

Within the feature fusion module, the network architecture is structured upon the Transformer encoder design, which includes both an attention layer and a feed-forward neural network (FFN). The fundamental components of the Transformer encoder include the Self-Attention Mechanism and Positional Encoding. The Self-Attention Mechanism enables the model to simultaneously focus on information from different positions in the input sequence, facilitating a better understanding of contextual relationships. Positional Encoding aids the model in processing the sequential information of the input, allowing it to comprehend the positional relationships within the sequence. In addition to these basic components, the Transformer incorporates features such as Multi-Head Attention, Residual Connections, Layer Normalization, and an FFN, also called a Multi-Layer Perceptron (MLP). Multi-Head Attention enables the model to parallelize attention across different subspaces, thereby enhancing its expressive power. Residual Connections play a crucial role in mitigating the vanishing gradient problem, ensuring more stable and efficient training. Layer Normalization contributes to stabilizing the training process by normalizing activations. The FFN module, present in each encoder and decoder layer, introduces non-linear transformations to the hidden representations at each position, further boosting the model’s capacity for non-linear feature learning.

In the feature fusion model’s architecture, the attention layer is specifically engineered to capture non-local correlations among various line segments.

(12) $$Att\left(f\right)=softmax\left(\frac{Q\left(f\right)\cdot K{\left(f\right)}^{T}}{\sqrt{d_{k}}}\right)\cdot V\left(f\right),$$

Within this context, linear layers, denoted as *Q*, *K*, and *V*, are instrumental in the extraction of query, key, and value vectors from the feature vector *f* associated with the given line segment. In this context, $d_{k}$ denotes the dimension of K(f), which is essential for normalization to prevent any bias in the attention scores due to key vector magnitudes. The FFN, denoted as F(·), is composed of a fully connected layer and incorporates a residual connection. As the final clustering results should not be influenced by the input order of the line segments, traditional positional embeddings used in Transformers are not employed. Consequently, the network architecture utilized within the feature fusion module can be summarized as follows:

(13) T(f)=F(Att(f)+f).

### 4.4. Losses and Vanishing Points Estimation

Utilizing the three unidentified vanishing points within the Manhattan world, we employ the cross-entropy loss function to categorize the line segments sequentially, starting with upward, followed by left and right directions in the horizontal orientation. The presence of the line segment classification loss is crucial for expediting and stabilizing the convergence of our approach, and its mathematical representation is as follows:

(14) $$L_{class}=-\frac{1}{M_{in}}\sum_{i=1}^{M_{in}}\sum_{c=1}^{3}g_{i}^{c}\log p_{i}^{c},$$

where $M_{in}$ is used here to represent the number of inlier line segments, with no consideration for outliers in the context of this loss function. Furthermore, $p_{i}^{c}$ is indicative of the forecasted probability that the *i*-th line segment is associated with the *c*-th group, while $g_{i}^{c}$ corresponds to the ground-truth label.

To handle outliers, we employ the binary cross-entropy loss function (BCE loss) to supervise $T_{score}$, which is represented as:

(15) $$L_{score}=-\frac{1}{M}\sum_{i=1}^{M}\left(y_{i}\log s_{i}+\left(1-y_{i}\right)\log\left(1-s_{i}\right)\right),$$

where *M* represents the total count of both inliers and outliers. In this context, outliers are defined as lines that might not contribute to the estimation of vanishing points. The variables $s_{i}$ and $y_{i}$, respectively, stand for the predicted score and the ground-truth of the *i*-th line segment. The total loss is the sum of the above two loss terms and can be represented as:

(16) $$L_{total}=L_{class}+L_{score}.$$

SVD is employed in our methodology to calculate vanishing points, with separate SVD computations conducted for each of the three groups of segmented lines represented as $L\in R^{M\times 3}$. In the pursuit of heightened result accuracy, we introduce a post-processing step based on RANSAC, conducting 10 iterations per vanishing point, before resorting to the SVD.

## 5. Experimental Results

### 5.1. Experiment Setup

**Figure 6:**
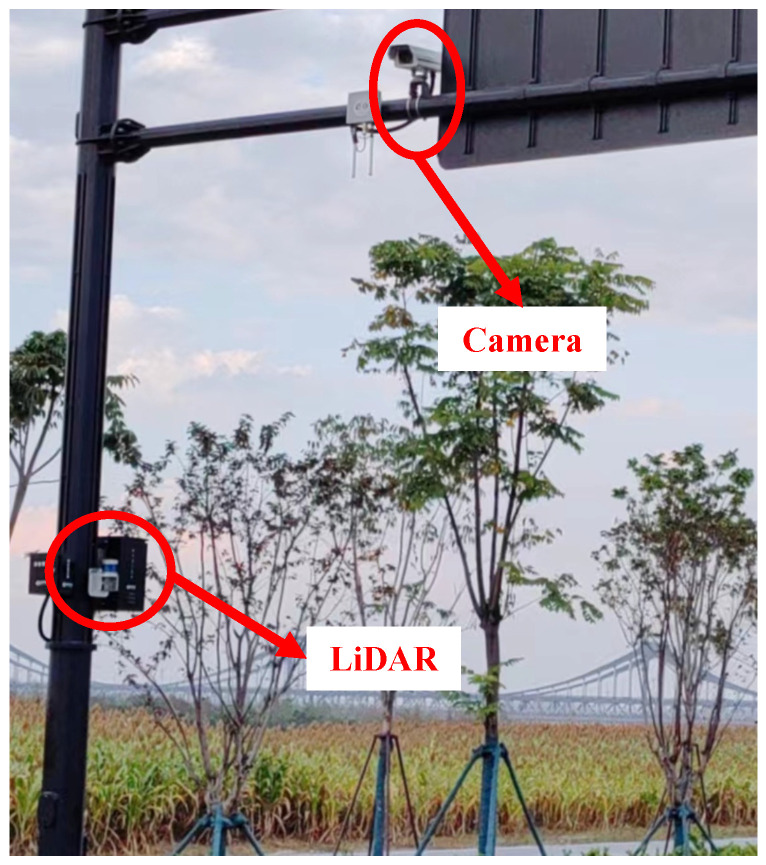
Data collection scenes: LiDAR and camera.

To validate the accuracy of the calibration model proposed in this paper, a dataset obtained from three roadside cameras and LiDAR sensors is used for evaluation. As shown in Figure 6, The LiDAR and camera have already been calibrated, and the perception results of the same vehicle in their respective sensors have been associated. This paper does not utilize LiDAR point cloud information but instead directly utilizes the speed detection results obtained from its embedded positioning functionality. Monocular speed estimation, akin to the method outlined in [58], utilizes accurate speed data obtained from LiDAR ground truth, in conjunction with the known camera height.

The vanishing point estimation model was trained separately on both the SU3 [59] and the ScanNet [60]. Both datasets conform to the Manhattan world assumption, where there should be three orthogonal vanishing points in each image. SU3 dataset comprises over 20,000 synthetic images from a panoramic dataset. In contrast, ScanNet originates from real-world indoor scenes. We follow [39,40] to obtain the ground truth of vanishing points. Our training and evaluation procedures are executed within the PyTorch framework. For training purposes, we employ SGD optimizer. The learning rate is deliberately set at 0.005, with the momentum and weight decay parameters finely tuned to 0.9 and 0.0001, respectively. For obtaining more accurate results, we also run a RANSAC based post-processing for 10 iterations per vanishing point before using SVD.

### 5.2. Error Metric for Comparison to Other Methods

To corroborate the efficiency of the approach postulated in this paper, speed estimation is utilized to gauge the precision of camera calibration. The quantification of speed adheres strictly to the methodology advocated by Sochor et al. [12], as outlined in their influential work on traffic speed measurement. In the scenario involving a monitored vehicle featuring reference points denoted by $p_{i}$ and associated timestamps $t_{i}$ for each reference point, where *i* varies from 1 to N, the speed *v* is determined via the application of Equation (17). This determination entails projecting the reference points $p_{i}$ onto the ground plane, represented as $P_{i}$, and such projection can be computed employing Equation (5).

The speed is determined by taking the median of speed values computed between successive temporal intervals. However, for the sake of measurement stability, it is recommended to avoid using the immediately following frame and, instead, select a time position that is spaced several video frames apart. The regulation of this temporal spacing is governed by the constant τ, and in all our experimental cases, we consistently set τ = 5 (resulting in an average time difference of 0.2 s). The evaluation of speed estimation was conducted in a real-world context, encompassing diverse scenarios, and further validated using the BrnoCompSpeed dataset [5]. This dataset comprises an extensive collection of over 20k vehicles, each accompanied by meticulously measured ground truth speed data obtained from various geographical locations. Scale inference is not within the scope of this paper; therefore, in the dataset, we directly utilize the true scale λ from BrnoCompSpeed for comparison.

(17) $$v={\mathrm{median}}_{i=1\ldots N-\tau }\left(\frac{\lambda_{reg}^{*}∥P_{i+\tau }-P_{i}∥}{t_{i+\tau }-t_{i}}\right)$$

### 5.3. Speed Error Results in Three Standard Experimental Traffic Scenes

**Figure 7:**
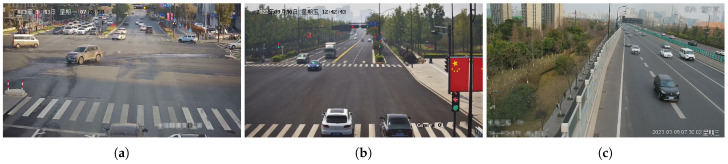
Standard experimental traffic scenarios: (**a**) Scene 1, (**b**) Scene 2, and (**c**) Scene 3., which all come from typical urban traffic scenarios with clear vanishing point cues. Scene 1 and Scene 2 are from two crossroads, while scene 3 is from a regular highway segment.

The speed measurement itself is the most crucial part of the evaluation. To validate the effectiveness of the method, three videos were captured in different scenarios in Hangzhou, as shown in Figure 7. The cameras had varying heights, angles, and positions, with a resolution of 1920 × 1080 and a frame rate of 25 FPS for each. Scenario one and two belong to typical four-way intersections, while scenario three represents a typical high-speed road intersection scene. DubskaCalib, SochorCalib, and DeepVPCalib are aliases for the methods proposed by Dubska in [11], Sochor in [12], and Kocur in [14], respectively. A consistent vehicle detection and tracking system was uniformly utilized throughout all experimental trials, ensuring the direct comparability of outcomes across different calibration scenarios.

**Figure 8:**
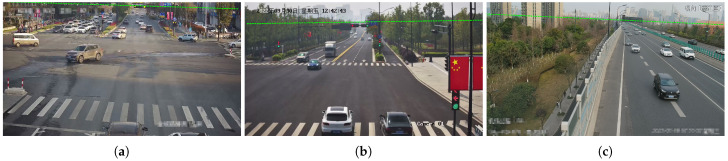
Horizontal line estimation results in three scenarios: (**a**) Scene 1, (**b**) Scene 2, and (**c**) Scene 3. The horizontal line can be obtained from the first two vanishing points.

As shown in Figure 8a, the horizontal line accurately reflect the camera’s rotation and are in close agreement with human perceptual results. To assess camera parameters with greater precision, we employ LiDAR values as an indirect measure of camera calibration accuracy.

#### 5.3.1. The Scene1 Results

**Figure 9:**
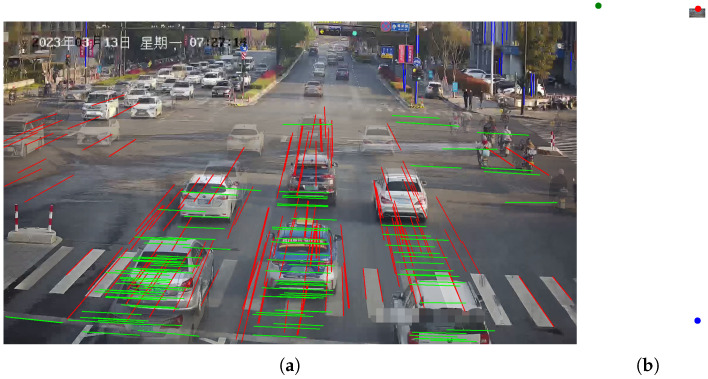
Line segments clustering results and vanishing point detection results in Scenario 1: (**a**) line segments clustering results and (**b**) vanishing point detection results.

**Figure 10:**
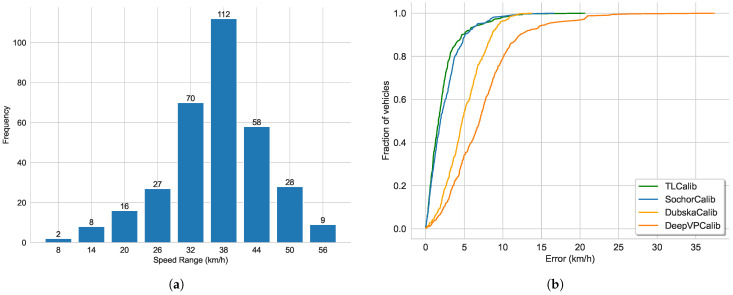
Speed distribution and cumulative speed estimation error results in Scenario 1: (**a**) speed distribution (km/h) and (**b**) cumulative speed estimation error.

As shown in Figure 9, we present our line clustering results and vanishing point detection results. We should be aware that the clustering results of the line segments in the images are extracted directly from the network outputs, without the need for post-processing procedures. It can be observed that the results of line segment classification closely align with human perceptual outcomes in Figure 9a. Furthermore, as depicted in Figure 9b, the vanishing point positions fall within a reasonable range. We also display the speed distribution and corresponding cumulative errors. In Scenario 1, we collected GNSS data ranging from 34 to 138 m as shown in Figure 10a, including velocity values for each frame, with speed distribution ranging from 7.2 to 61.2 km/h. As shown in Figure 10b, it can be observed that our method closely approaches SochorCalib [12], surpasses DubskaCalib [52], and outperforms previous heatmap-based methods [14]. DubskaCalib exhibits significant errors, partly due to disturbances such as motion blur and shadows in the scene, which make it less stable in estimating the second vanishing point. The results also indicate that our approach has advantages in vehicle localization and speed estimation and can be applied to a wider range of real-world scenarios.

#### 5.3.2. The Scene2 Results

**Figure 11:**
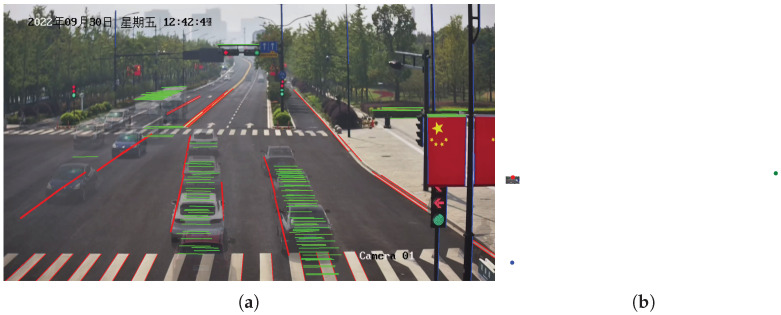
Line segments clustering results and vanishing point detection results in Scenario 2: (**a**) line segments clustering results and (**b**) vanishing point detection results.

**Figure 12:**
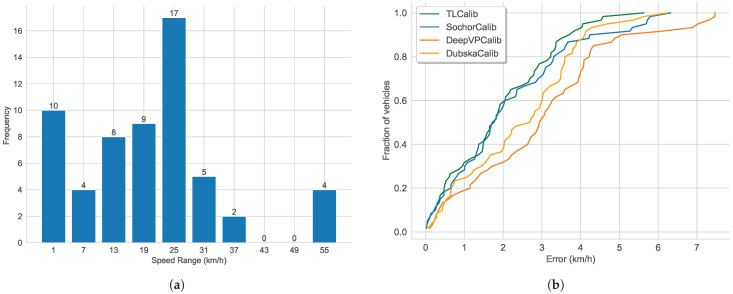
Speed distribution and cumulative speed estimation error results in Scenario 2: (**a**) speed distribution (km/h) and (**b**) cumulative speed estimation error.

As shown in Figure 11, the line clustering results and vanishing point detection results are similar to those in Figure 9. However, the position of the second vanishing point in Figure 11b is different from Figure 9b, indicating a slight variation in the tilt direction. In contrast to Scenario 1, Scenario 2 involved collecting GNSS data ranging from 29 to 107 m, with fewer data points compared to Scenario 1 but a similar distance distribution. As shown in Figure 12a, the speed distribution in this scenario ranges from 1.8 to 57.6 km/h. Looking at Figure 12b, it is evident that our method and SochorCalib [12] achieve competitive accuracy, once again confirming the reliability and effectiveness of our approach. Due to differences in data distribution between the two scenarios, there are slight variations in the results, but the relative accuracy trends among the methods remain consistent. While DubskaCalib is still not on par with our method and SochorCalib, its precision is notably higher than in Scenario 1. This could be attributed to the fact that Scenario 2 has fewer vehicles, clearer and more stable edges, and is less affected by motion blur. In Scenario 2, the accuracy of DubskaCalib may have been somewhat affected by the position of the second vanishing point due to scene-specific reasons. Another contributing factor could be that in Scenario 1, the data has a greater maximum distance than in Scenario 2. Larger distances lead to increased inter-frame positioning errors for vehicles, consequently resulting in larger speed estimation errors. Compared to DubskaCalib, SochorCalib appears to be more stable, and our method’s accuracy remains relatively unaffected by the specific scene conditions. We believe this is mainly due to our approach’s combination of scene cues and vehicle cues, where the integration of semantic and geometric cues enhances the model’s robustness.

#### 5.3.3. The Scene3 Results

**Figure 13:**
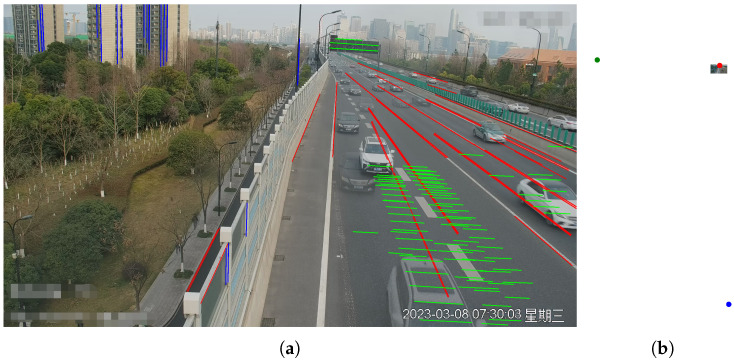
Line segments clustering results and vanishing point detection results in Scenario 3: (**a**) line segments clustering results and (**b**) vanishing point detection results.

**Figure 14:**
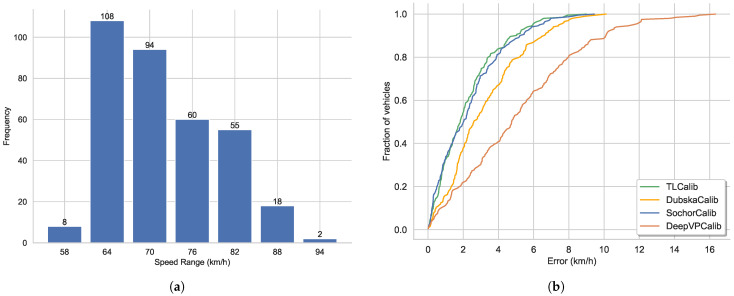
Speed distribution and cumulative speed estimation error results in Scenario 3: (**a**) speed distribution (km/h) and (**b**) cumulative speed estimation error.

Similarly, in Figure 13, we present the model’s line clustering results and vanishing point detection results at a expressway. It can be observed that in expressway scenarios, there are more lane markings and vehicles, resulting in more abundant and distinct line markings. Additionally, road lamps and road edges can be utilized as features to infer the direction of the third vanishing point. Scenario 3 differs from the previous two scenarios in several ways, where the distance distribution ranges from 29 to 187 m, and the speed distribution ranges from 57.6 to 100.8 km/h as shown in Figure 14a, representing a typical expressway scenario that is significantly different from the previous two. It is worth noting that, despite the larger speed distribution in the expressway scenario compared to the previous two scenarios, the errors are nearly similar. Our method continues to outperform the other three methods as shown in Figure 14b. The inclusion of Scene 3 in our evaluation allows us to assess the model’s robustness across different scenarios with varying speed distributions. Despite the significant differences in the scene characteristics and speed ranges, our method consistently maintains a high level of accuracy, demonstrating its effectiveness in diverse real-world scenarios. In Scenario 3, some lane markings and line segments around the surrounding buildings are very clear, which contributes to the model’s higher accuracy.

### 5.4. Further Comparison of the Results of More Complex Traffic Scenarios

**Figure 15:**
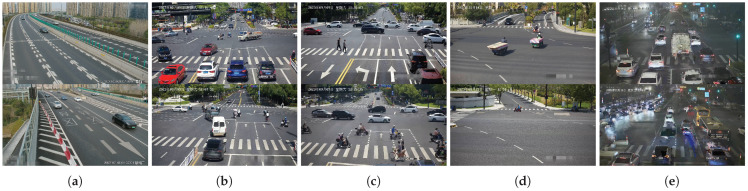
More complex traffic scenarios (**a**–**e**). These scenarios represent situations where vanishing point clues gradually decrease.

The previous approach was generally applicable to scenes where clear clues were available for inferring vanishing points. The experiments in Section 5.3, which precedes the current discussion, were primarily conducted in such scenarios. However, the complexity of traffic scenes, as illustrated in the five scenarios from (a) to (e) in Figure 15, poses additional challenges. To further validate our method and compare it with others in more complex scenes, we conducted additional speed error accuracy comparisons in five scenarios, as shown in Figure 15. The vehicle trajectories in these five scenarios gradually diminish. For instance, scenario a includes partial straight-line trajectories but also involves curves. In scenario b, the vehicle trajectories are not straight. Scenario c lacks the first vanishing point clues but includes clues related to the second vanishing point. Scenario d represents scenes with less obvious vehicle trajectories or scenes with sparse traffic flow. Scenario e illustrates nighttime video streaming, where network issues may lead to stream instability, resulting in video jitter or interruptions.

**Figure 16:**
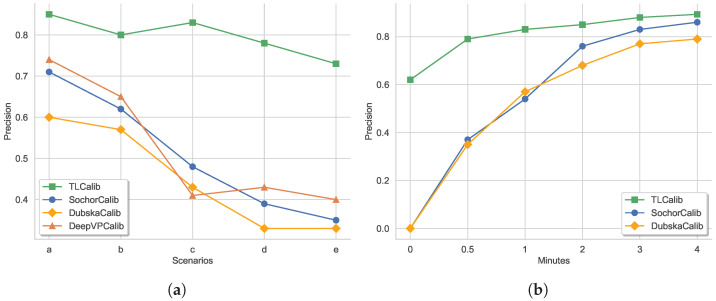
(**a**) Speed estimation accuracy of different methods in five scenarios; (**b**) speed estimation accuracy of different methods at various cumulative sampling times.

The results from Figure 16a indicate that, for scenes containing partially curved trajectories like scenario a, SochorCalib exhibits acceptable accuracy, but it is not as effective as DeepVPCalib. This may be because DeepVPCalib, which is based on a deep learning approach for detecting vanishing points’ heatmaps, has fewer limitations in curved scenarios. The results for scenario b are similar to scenario a, but the overall accuracy is lower for all four methods. This is because scenario a includes a significant number of straight trajectories. Regarding scenario c, it can be observed that in scenes lacking the trajectory towards the same direction as the camera orientation, the performance of the other three methods sharply declines, while our method maintains relatively high accuracy. This is largely attributed to the fact that our method is not strictly dependent on vehicle trajectories. The results for scenario d also support this point. As for scenario e, our method experiences a certain degree of decline in performance, but it still maintains high accuracy for other methods.

### 5.5. The Speed Errors in Different Sampling Time

The inference process of camera calibration for the compared methods in the experiment relies on tracking feature points over a certain duration. Additionally, the optical flow tracking results are utilized in the voting process within the diamond space to determine the final vanishing point position for camera calibration, and different tracking durations can lead to varying estimates of vanishing points. Therefore, we conducted further experiments to examine the impact of cumulative tracking duration (or sampling time) on the final calibration accuracy. We selected six time moments for sampling, and the results are shown in Figure 16b. Since DeepVPCalib does not strictly depend on longer video durations for inference, we mainly compared our method with SochorCalib and DubskaCalib. It can be observed that our method produces relatively acceptable results from the beginning, even though they are less accurate. At the 0.5 min and 1 min marks, SochorCalib and DubskaCalib show a noticeable enhancement in accuracy; however, there remains a substantial disparity compared to the accuracy achieved by our method. By the 4 min mark, SochorCalib and DubskaCalib achieve competitive results with our method. The results clearly indicate that our method converges at a faster rate, and of course, longer inference times lead to better precision enhancement.

### 5.6. Comparison to Other Calibration Methods on Public Dataset

Since the primary objective of this work is to assess camera calibration accuracy, and the methods employed do not estimate scale, this paper does not consider scale issues. To ensure a rigorous and accurate assessment of our accuracy, we also conducted evaluations and comparisons on publicly available datasets and ours datasets, using known camera mounting heights as scene scale [58]. Since the true camera parameters in our scene are unknown, by calculating the error between camera positioning and the actual LiDAR positioning, we optimized the camera parameters, considering them as the ground truth camera parameters. Our results include three metrics: mean, median, and 99%, and comparisons were made on our dataset and the BrnoCompSpeed dataset. From Table 1, we compared our approach with three other methods, it can be observed that whether on BrnoCompSpeed or in our own scenarios, our method outperforms traditional roadside auto camera calibration algorithms like SochorCalib, as well as current deep learning-based methods. Furthermore, our results on SU3 are better than those trained on ScanNet, primarily because the scenes in SU3 more closely resemble real traffic scenarios, whereas ScanNet is more focused on indoor scenes.

### 5.7. Ablation Study

To validate the significant role of traffic flow cues in camera calibration, we conducted ablation experiments on our model. From Table 2, it is evident that we evaluated the model’s performance separately for dynamic traffic flow and for static scenes. Experimental results indicate that camera calibration accuracy obtained solely based on optical flow is higher than that in static traffic scenes. When combining both optical flow and scene features, the model’s accuracy further improves. This demonstrates that relying solely on static traffic scenes for calibration without considering vehicle motion cues cannot accurately estimate the second vanishing point, which subsequently reduces the accuracy of downstream tasks such as speed estimation. This effect is particularly pronounced in scenes with fewer line segments in the direction of the second vanishing point. Information from both the scene and traffic flow is crucial for camera calibration in traffic scenarios, and combining them results in higher accuracy. So, both sources of information are indispensable.

## 6. Conclusions

In this paper, we propose a Transformer-based self-calibration method for roadside cameras called “TLCalib”. Our approach integrates traffic scene features and vehicle characteristics, combining the geometric representation of line segments in the road scene with their surrounding semantic features for vanishing point detection. Moreover, leveraging the advantages of the Transformer architecture, the three vanishing point features are unifiedly processed in the line feature fusion module. This design not only addresses the limitations of traditional camera self-calibration methods that rely on manually designed edge-background models but also simplifies the steps involved in vanishing point estimation, reducing the need for manual parameter design. Furthermore, it enhances the generalization capability of deep learning-based camera calibration methods in real-world scenarios, allowing for more comprehensive feature extraction from the three vanishing point directions and, consequently, improving the accuracy of camera calibration.

The method presented in this paper did not address the issue of scale estimation. In the future, we will investigate how to integrate scale estimation into the model. We also considered domain shift issues, but the decrease in accuracy due to domain shift is not significant in our scenario. This is likely because a significant portion of the extracted line segment features is common across multiple domains. Line detection results share similar features and attributes, and the use of geometric priors is data-efficient since this knowledge no longer needs to be acquired from the data. As a result, the model possesses a degree of generalization capability to meet basic roadside 3D application requirements. However, it is generally necessary to perform fine-tuning directly in traffic scenarios for higher accuracy, which we currently lack the sufficient data for. Traditional vanishing point estimation methods are entirely based on prior knowledge and do not require consideration of domain shift issues, as they also do not leverage semantic features in images. Therefore, in the future, we plan to further enhance the model’s domain adaptation capabilities, possibly by combining some traditional methods, while simultaneously improving model accuracy.

## Figures and Tables

**Table 1:** Comparative analysis of dataset results (speed estimation errors in km/h).

	BrnoCompSpeed			Ours		
Method	Mean	Median	99%	Mean	Median	99%
DubskaCalib [11]	8.59	8.76	14.78	5.06	4.75	13.73
SochorCalib [12]	3.88	3.35	5.47	2.46	2.30	6.24
DeepVPCalib [14]	13.96	10.58	20.75	8.23	7.63	19.01
TLCalib-ScanNet	4.54	4.01	9.91	3.13	2.96	7.44
TLCalib-SU3	3.61	3.67	8.55	2.29	1.66	5.95
GT	-	-	-	1.58	1.62	4.25

**Table 2:** Speed estimation results in different scenes (speed estimation errors in km/h).

Scene	Mean	Median	99%
Static Traffic Scene Only	6.72	6.12	12.54
Dynamic Optical Flow	3.48	3.32	7.43
Static Scene + Dynamic Optical Flow	2.81	2.36	4.55

## Data Availability

The data presented in this study are available on request from the corresponding author. The data are not publicly available due to privacy or ethical restrictions.

## Abbreviations

The following abbreviations are used in this manuscript:

PTZ	pan-till-zoom
CCS	camera coordinate system
WCS	world coordinate system
ICS	image coordinate system
SGD	stochastic gradient descent
SVD	singular value decomposition
RANSAC	random sample consensus
km/h	kilometers per hour
